# Association of major dietary patterns with advanced glycation end products and high-sensitivity C-reactive protein in people with type 1 diabetes mellitus

**DOI:** 10.1186/s12937-023-00860-x

**Published:** 2023-07-26

**Authors:** Zohreh Ebrahimi, Zahra Shojaeian, Fatemehsadat Amiri, Ahmad Esmaillzadeh, Omid Sadeghi, Alireza Esteghamati, Seyed Adel Jahed, Sara Sedaghat

**Affiliations:** 1 Department of Nutrition, School of Public Health, Iran University of Medical Sciences, Tehran, Iran; 2 Department of Community Nutrition, School of Nutritional Sciences & Dietetics, Tehran University of Medical Sciences, Tehran, Iran; 3 Nutrition and Food Security Research Center, Department of Community Nutrition, Student Research Committee, School of Nutrition and Food Science, Isfahan University of Medical Sciences, Isfahan, Iran; 4 Endocrinology and Metabolism Research Center (EMRC), Internal Medicine Department, Vali-Asr Hospital, Tehran University of Medical Sciences, Tehran, Iran; 5 Gabric Diabetes Education Association, Tehran, Iran

**Keywords:** Type 1 diabetes, Dietary pattern, AGEs, hs-CRP

## Abstract

**Background:**

The associations of diet with serum levels of advanced glycation end products (AGEs) and high-sensitivity C-reactive protein (hs-CRP) have been examined in patients with type 2 diabetes mellitus (T2DM). However, data on patients with type 1 diabetes mellitus (T1DM) are limited. Therefore, this study was conducted to investigate the associations of major dietary patterns with serum levels of AGEs and hs-CRP among patients with T1DM.

**Methods:**

A total of 229 patients with T1DM participated in this current cross-sectional study. We collected dietary data using a validated food frequency questionnaire (FFQ). The factor analysis approach was used to determine major dietary patterns. A fasting blood sample was collected from each participant to assess serum levels of AGEs and hs-CRP. The associations of dietary patterns with elevated levels of AGEs and hs-CRP were assessed using binary logistic regression.

**Results:**

Patients with T1DM in the highest tertile of a Western dietary pattern had 4.32 times higher odds of having elevated AGEs than those in the lowest tertile (OR: 4.32, 95% CI: 1.86–10.05). Additionally, adherence to the Western diet was associated with 2.97 times greater odds of having elevated hs-CRP (> 3 mg/L) (OR: 2.97, 95% CI: 1.22–7.24) in these patients. Such positive associations were not observed for unhealthy and traditional dietary patterns. Moreover, higher adherence to a semi-healthy diet (characterized by high consumption of white meat, whole grains, processed meat, and a low salt intake) was associated with 87% lower odds of having elevated hs-CRP (OR: 0.13, 95% CI: 0.05–0.35). However, we found no significant association between the semi-healthy diet and AGEs levels.

**Conclusion:**

We found that adherence to a Western dietary pattern was associated with elevated levels of AGEs/hs-CRP in patients with T1DM. Also, we discovered a significant inverse association between adherence to a semi-healthy diet and hs-CRP levels.

**Supplementary Information:**

The online version contains supplementary material available at 10.1186/s12937-023-00860-x.

## Introduction

Type 1 diabetes mellitus (T1DM), with an annual incidence of 3–4%, is increasing worldwide [1]. Uncontrolled diabetes may cause acute and long-term complications, which cause increased morbidity and mortality [2]. In addition to abnormal glucose hemostasis and hyperlipidemia, increased levels of advanced glycation end products (AGEs) may contribute to the etiology of diabetes complications [3]. Previous studies have shown that increased levels of AGEs are associated with elevated levels of inflammatory biomarkers and oxidative stress [4]. In addition, AGEs contribute to various microvascular and macrovascular complications by forming cross-links between molecules in the basement membrane of the extracellular matrix and by engaging the receptor for advanced glycation end products (RAGE) [5].

AGEs are formed by irreversible non-enzymatic reactions between reducing sugars, such as glucose and fructose, and amino groups in proteins, lipids, and nucleic acids [6]. Therefore, uncontrolled hyperglycemia increases the production of AGEs in T1DM patients due to hyperglycemic environments, and diet can affect the production of endogenous AGEs by influencing glucose homeostasis. Prior studies suggested that Western foods, such as grilled and broiled meats and french fries, contain high amounts of pro-inflammatory AGEs [7]. However, recent studies concluded that the increasing effect of these foods on AGEs levels is not higher than other foods [8, 9]. In addition, there is a lack of consistent evidence to support the assertion that dietary AGEs are significant sources of pro-inflammatory AGEs [7].

High-sensitivity C-reactive protein (hs-CRP) is one of the well-recognized markers for inflammation and has been associated with many complications and chronic diseases among patients with diabetes [10]. Diet can affect serum levels of inflammatory biomarkers [11]. Previous studies suggested that adherence to a diet rich in vegetables and fruits, whole grains, nuts, and plant oils, with a low amount of meat, sweetened drinks, processed foods, and solid fats was associated with lower hs-CRP levels [10, 12]. However, few studies have examined the association between diet and hs-CRP levels among patients with T1DM.

Previous studies on the association between diet and serum levels of AGEs and hs-CRP have mainly focused on non-diabetic patients or patients with T2DM. Therefore, little attention has been paid to patients with T1DM. Also, most studies assessed the intakes of individual nutrients, foods, and food groups in relation to AGEs and hs-CRP levels, while data on dietary patterns are scarce. The dietary pattern approach in assessing diet-disease relations is better than focusing on a single food or nutrient intake due to decreasing the co-linearity problem which might occur when assessing single food and nutrient intakes [10, 13]. Given the importance of AGEs and hs-CRP levels in patients with T1DM and the influence of diet on these biomarkers, this study was conducted to assess the associations of major dietary patterns with serum levels of AGEs and hs-CRP in patients with T1DM.

## Materials and methods

### Study design and participants

This cross-sectional study was done on patients with T1DM who were referred to the Gabric Diabetes Education Association in Tehran, Iran, diabetes clinics in Tehran city, and Imam Khomeini hospital in Tehran, Iran. We included patients if they (1) had T1DM for ≥ 1 year, (2) had an age range of ≥ 18 years, and (3) were treated with insulin. Also, we excluded patients who had a severe hypoglycemic episode (requiring assistance) during the last 3 months, pregnant women, patients with severe diabetes complications (advanced microvascular and macrovascular complications), those who experienced hyperglycemia-induced ketoacidosis in the last month, and patients with other autoimmune diseases such as celiac disease. We first included 252 patients with T1DM based on the inclusion and exclusion criteria. Participants were invited to the Nutrition Clinic of Iran University of Medical Sciences, Tehran, Iran, for data collection. Trained interviewers gathered the required information on socio-demographic characteristics, physical activity, and dietary intakes. In addition, participants were asked to arrive in a fasting state for the collection of biological samples. After collecting data and excluding patients with incomplete data and those who had reported energy intake out of 800–4200 kcal [14], 229 patients were included in the current analysis. The study protocol was approved by the ethics committee of the Iran University of Medical Sciences, Tehran, Iran (IR.IUMS.REC 1395.9411323001). The written informed consent form was gathered from each participant.

### Sample size calculation

In the current study, we calculated the required sample size by considering the type 1 error of 5% (α = 0.05) and the difference between diabetic patients and healthy individuals in terms of AGEs levels based on the study of Kalousová et al., in which AGEs levels in diabetic patients were 24% higher than healthy individuals [15]. The following formula was used for sample size calculation:

1 $$n = \frac{{{{({Z_{1 - \alpha }})}^2}}}{d} \times P(1 - P)$$

α = conventional multiplier for alpha = 0.05 that was 1.96.

P = The difference between diabetic patients and healthy individuals in terms of AGEs levels based on Kalousová et al. study [15]: 24%.

d = the accuracy of estimation which was considered 25% of P in the current study: 0.06.

Based on this formula, we required 195 participants in the current cross-sectional study. However, data from 229 participants were included in the current analysis.

### Dietary intake assessment

Dietary intakes were assessed using a validated block-format 168-item food frequency questionnaire (FFQ). This questionnaire was designed specifically for Iranian adults [16]. Also, previous studies on diet-disease associations confirmed the reliability and validity of the questionnaire [17–19]. Two experienced interviewers fulfilled FFQ for each participant. Based on this questionnaire, participants were asked to report their dietary intakes in a day, week, month, or year format. Moreover, they reported their intakes based on the serving size of each food item. To increase the estimates’ accuracy, interviewers showed household measures or serving sizes of each food item to participants [20]. Finally, based on the consumption frequency of each item and serving size, we calculated the gr/day intake for each food item. In addition, we calculated the daily nutrient intake for each participant according to the nutrient content of all foods. The US Department of Agriculture’s national nutrient databank was used to obtain the nutrient content of foods [21].

### Construction of dietary patterns

We first merged the food items (in FFQ) to obtain 19 food groups for the construction of major dietary patterns (Supplementary Table 1). This combination was done based on the similarity of the nutrient content of food items [22]. Four food items, including potato, mayonnaise sauce, salt, and tea/coffee were considered individually in dietary pattern construction because of their unique nutrient contents or their contribution to a specific dietary pattern (Supplementary Table 1). In total, 23 food groups or items were included in the factor analysis to identify major dietary patterns. We performed the factor analysis with the orthogonal transformation (varimax procedure). Obtained factors were retained for additional analysis based on Eigen values obtained in the scree plot. In the current study, we kept factors with Eigen values of ≥ 1.35 to determine major dietary patterns. The factor score for each pattern was calculated by summing the intakes of food groups or items weighted by their factor loadings, and each participant received a factor score for each identified pattern. A higher score for each pattern was associated with greater adherence to that pattern.

### Anthropometric and biochemical assessment

Weight was measured with minimum clothing, without shoes, using a digital scale with an accuracy of 100 g. Standing height was measured using a stadiometer with an accuracy of 0.5 cm without shoes, flat feet against a wall, and arms at the sides [23]. BMI was calculated as weight (kg)/ height (${m}^{2}$).

A 12 ml fasting blood sample was collected from each individual for biochemical assessment. Then, blood was centrifuged (RPM: 4000 and time: 10 min), fractioned into different aliquots, and stored in the freezer (–80 °C). Serum concentrations of AGEs were determined using the ELISA Kit (ZellBio GmbH, Germany) with an intra-assay CV of < 10% and an inter-assay CV of < 12%. Serum levels of hs-CRP were measured by the immunoturbidimetric method (Pars Azmoon, Tehran, Iran). In the current study, we considered the third tertile of AGEs levels as elevated AGEs. Furthermore, the hs-CRP concentrations of ≥ 3 mg/L were considered elevated hs-CRP. Based on multiple epidemiological and intervention studies and also according to the American Heart Association, different levels of hs-CRP have been associated with future major cardiovascular risk (hs-CRP < 1 mg/L = low risk; 1–3 mg/L = intermediate risk; 3–10 mg/L = high risk; >10 mg/L = unspecific elevation) among diabetic patients [24, 25]. Since the high risk of cardiovascular disease was important in the current study, we considered hs-CRP ≥ 3 mg/L as high hs-CRP.

### Assessment of other variables

Data on age, gender (male/female), education (university graduated/ non-university education), marital status (married/single), smoking (never or ex-smoker/current smoker), DM-related training (yes/ no), DM duration (> 10 y/ 10 > y), and drug use (yes/no) were collected from each participant through a face-to-face interview using a research-made questionnaire. Furthermore, we used the International Physical Activity Questionnaire (IPAQ) to assess physical activity. All results of the IPAQ were expressed as metabolic equivalents per week (MET-h/week). This questionnaire was previously validated for the Iranian population [26].

### Statistical analyses

To evaluate the association between adherence to dietary patterns and outcome variables, we first categorized individuals based on the tertiles of dietary patterns’ scores. Then, the one-way analysis of variance (ANOVA) was applied to examine the differences in continuous variables across tertiles of dietary patterns. In addition, the distribution of participants in terms of categorical variables across the tertiles of dietary patterns’ scores was assessed using the Chi-square test. We used binary logistic regression in different adjusted models to evaluate the associations of dietary patterns with elevated levels of AGEs and hs-CRP. First, we included age (continuous), gender (male/female), and energy intake (continuous) in model 1 to assess whether the obtained associations were mediated by these variables or not. In the second model, we additionally adjusted for smoking (never or ex-smoker/current smoker), duration of diabetes (≥ 10 y/<10 y), DM-related training (yes/no), physical activity (continuous), and drug use (yes/no) because these variables might be associated with dietary intakes and AGEs/hs-CRP levels. In the last model, further adjustment was made for BMI (continuous) to evaluate the influence of obesity on obtained associations. In all analyses, individuals in the first tertile of dietary patterns’ scores were considered as a reference group. We treated the tertile categories as an ordinal variable in the binary logistic regression to evaluate the overall trend of odds ratios across increasing tertiles of dietary patterns’ scores. All analyses were performed using the statistical software package IBM SPSS Statistics version 18. P-values were considered significant at < 0.05.

## Results

The mean age of study participants was 32.31 ± 10.96 years, and 61.7% were female. In addition, 27.4% of patients with T1DM had high levels of hs-CRP (> 3 mg/L). We identified four dietary patterns based on the factor analysis (Table 1). The first dietary pattern, the “Western” dietary pattern, was characterized by high consumption of snacks, potatoes, sweets and desserts, pizza, sweetened drinks, and egg. The second pattern was an “unhealthy” dietary pattern with a high content of red and organ meats, refined grains, solid fats, and a low amount of fruits, vegetables, legumes, and low-fat dairy products. The “traditional” dietary pattern was associated with high intakes of nuts, liquid oils, high-fat dairy products, mayonnaise sauce, and dried fruits. The last pattern was a “semi-healthy” dietary pattern characterized by high consumption of white meat, whole grains, processed meat, and a low intake of salt. The four dietary patterns explained 34.29% of the whole variance in dietary intakes.

**Table 1:** Food loading matrix for major dietary patterns

Food group	Dietary patterns			
	“Western”	“Unhealthy”	“Traditional”	“Semi-healthy”
Snack	0.765			
Potato	0.532			
Sweets and desserts	0.515			
Pizza	0.472			
Sweetened drinks	0.428			
Eggs	0.422			
Vegetables		− 0.596		
Legumes		− 0.559		
Low-fat dairy products		− 0.506		
Fruits		-0.468		
Refined grains		0.409		
Red meats & Organ meats		0.287		
Solid fats		0.212		
Dried fruits			0.608	
Liquid oils			0.551	
High-fat dairy products			0.457	
Mayonnaise sauce			0.410	
Nuts			0.349	
White meats				0.803
Whole grains				0.451
Processed meats				0.429
Salt				− 0.418
Tea and coffee		-	-	-
Variance explained (%)	10.02	9.16	7.58	7.52

The highest factor loadings values were remained for simplicity

Demographic characteristics of patients with T1DM across tertiles of dietary patterns’ scores are shown in Table 2. Compared with the lowest tertile of the Western dietary pattern scores, patients with T1DM in the highest tertile were younger and were less likely to be female and married. Moreover, there was a significant difference in terms of participants’ age and gender and diabetes duration across tertiles of unhealthy dietary pattern scores. Greater adherence to the traditional dietary pattern was associated with lower BMI and DM-related training. In addition, participants in the third tertile of the semi-healthy dietary pattern were more likely to be university educated and less likely to be female and current smokers compared with those in the first tertile. No other significant difference was observed in this regard.

**Table 2:** Demographic characteristics of participants across tertiles of dietary patterns

	“Western” dietary pattern					“Unhealthy” dietary pattern					“Traditional” dietary pattern					“Semi-healthy” dietary pattern			
	T1	T2	T3	P^*^		T1	T2	T3	P^*^		T1	T2	T3	P^*^		T1	T2	T3	P^*^
Age (y)	35.0 ± 12.4	30.9 ± 9.6	30.9 ± 10.2	0.03		34.2 ± 11.9	32.8 ± 10.4	29.8 ± 10.1	0.04		33.3 ± 11.4	32.5 ± 10.1	30.9 ± 11.2	0.39		32.2 ± 12.4	32.1 ± 10.7	32.5 ± 9.7	0.97
BMI (kg/m^2^)	24.9 ± 3.7	25.0 ± 3.8	24.3 ± 3.5	0.51		25.0 ± 3.9	24.9 ± 3.5	24.4 ± 3.6	0.66		24.6 ± 3.4	26.6 ± 4.2	24.1 ± 3.3	0.05		24.6 ± 3.6	24.8 ± 4.3	24.8 ± 3.1	0.92
PA (MET-h/wk)	454 ± 357	486 ± 495	508 ± 480	0.76		535 ± 508	472 ± 379	441 ± 446	0.42		507 ± 458	488 ± 443	453 ± 444	0.75		392 ± 324	528 ± 467	527 ± 519	0.10
HbA1c (%)	8.0 ± 1.5	7.9 ± 1.5	8.3 ± 1.6	0.28		8.1 ± 1.9	8.2 ± 1.4	8.0 ± 1.3	0.67		7.8 ± 1.4	8.2 ± 1.6	8.3 ± 1.6	0.22		8.3 ± 1.6	8.0 ± 1.7	8.0 ± 1.4	0.53
Daily insulin dose (unit)	50.7 ± 15.5	51.4 ± 18.6	55.5 ± 18.8	0.20		49.5 ± 14.4	50.0 ± 17.8	58.1 ± 19.6	0.003		53.4 ± 22.1	52.9 ± 15.9	51.3 ± 14.6	0.76		53.7 ± 19.6	51.3 ± 15.1	52.6 ± 18.5	0.71
Female %	71.1	67.5	47.4	< 0.01		63.2	74.0	48.7	< 0.01		59.2	71.4	55.3	0.10		69.7	71.4	44.7	< 0.01
Current smoker %	18.4	16.9	25.0	0.41		17.1	15.6	27.6	0.12		18.4	19.5	22.4	0.82		89.5	79.2	71.1	0.02
DM-related training (yes %)	85.5	80.5	85.5	0.62		88.2	84.4	78.9	0.30		93.4	81.8	76.3	0.01		76.3	89.6	85.5	0.07
Married %	56.6	35.1	38.2	0.01		42.1	49.4	38.2	0.36		40.8	48.1	40.8	0.57		43.4	44.2	42.1	0.96
University educated %	67.1	64.9	60.5	0.69		60.5	67.5	64.5	0.66		69.7	63.6	59.2	0.39		46.1	71.4	75.0	< 0.01
DM duration (> 10 y %)	72.4	63.6	71.1	0.45		78.9	68.8	59.2	0.03		71.1	76.6	59.2	0.06		69.7	71.4	65.8	0.74
Drug use (yes %) ^b^	28.9	26.0	15.8	0.13		23.7	27.3	19.7	0.54		26.3	26.0	18.4	0.43		27.6	27.3	15.8	0.14
Insulin type %				0.30					0.31					0.61					0.26
Lantus and NovoRapid	65.8	67.5	61.8			64.5	67.5	63.2			67.1	66.2	61.8			61.8	67.5	65.8	
NPH and Regular	25.0	13.0	26.3			17.1	22.1	25.0			18.4	20.8	25.0			27.6	19.5	17.1	
Lantus and Apidra	3.9	9.1	2.6			3.9	6.5	5.3			5.3	3.9	6.6			3.9	7.8	3.9	
Levemir and NovoRapid	5.3	3.9	5.3			7.9	0	6.6			5.3	6.5	2.6			2.6	2.6	9.2	

Data are presented as mean ± SD or percent

Abbreviation: BMI: body mass index, DM: diabetes mellitus, PA: physical activity

^*^Obtained from the analysis of variance (ANOVA) or chi-squared test, where appropriate

^b^ Considered as the use of antihypertensive medication, lipid lowering medication and Levothyroxine

The dietary intakes of participants across tertiles of dietary patterns’ scores are indicated in Table 3. Greater adherence to the Western dietary pattern was associated with higher intakes of red meat, low-fat dairy products, energy, protein, carbohydrate, fat, sodium, calcium, fiber, and a lower intake of nuts. Considering the unhealthy dietary pattern, participants in the top tertile had greater intakes of red meat and fat and lower intakes of fruits, vegetables, low-fat dairy products, nuts, energy, protein, sodium, calcium, and fiber compared with those in the bottom tertile. Individuals with a higher adherence to the traditional dietary pattern had greater intakes of fruits, vegetables, red meat, nuts, whole grains, energy, protein, carbohydrate, fat, sodium, calcium, and fiber. Also, a higher score for the semi-healthy dietary pattern was related to higher intakes of fruits, red meat, nuts, whole grains, energy, protein, carbohydrate, fat, sodium, calcium, and fiber. Other differences in dietary intakes among the tertiles of dietary patterns’ scores were not significant.

**Table 3:** Dietary intakes of selected food groups and nutrients across tertiles of dietary patterns

	“Western” dietary pattern					“Unhealthy” dietary pattern					“Traditional” dietary pattern					“Semi-healthy” dietary pattern			
	T1	T2	T3	P^*^		T1	T2	T3	P^*^		T1	T2	T3	P^*^		T1	T2	T3	P^*^
Food groups (g/d)																			
Fruits	432.4 ± 354	392.8 ± 417	413.9 ± 255	0.78		592.0 ± 509	338.8 ± 170	308.9 ± 175	< 0.01		286.2 ± 176	360.7 ± 268	592.6 ± 460	< 0.01		330.5 ± 222	348.0 ± 207	561.1 ± 492	< 0.01
Vegetables	562.4 ± 524	409.5 ± 284	521.6 ± 426	0.07		783.7 ± 580	408.6 ± 229	301.2 ± 177	< 0.01		383.8 ± 347	426.6 ± 289	682.8 ± 541	< 0.01		506.1 ± 452	441.7 ± 401	545.2 ± 422	0.31
Red meat	49.1 ± 35.0	63.5 ± 47.6	66.9 ± 53.4	0.04		44.8 ± 38.5	61.9 ± 42.8	72.9 ± 52.9	< 0.01		43.3 ± 30.5	60.5 ± 42.2	75.8 ± 57.3	< 0.01		47.7 ± 37.2	61.6 ± 51.8	70.4 ± 46.7	< 0.01
Low-fat dairy	233.9 ± 170	318.9 ± 323	333.4 ± 253	0.03		449.7 ± 354	276.1 ± 146	160.9 ± 122	< 0.01		342.1 ± 329	293.5 ± 209	251.0 ± 219	0.09		306.4 ± 322	285.0 ± 234	295.3 ± 213	0.87
Nuts	29.2 ± 62.7	13.0 ± 14.3	21.7 ± 14.9	0.03		31.0 ± 60.0	20.0 ± 24.9	12.9 ± 10.0	0.01		10.9 ± 6.9	21.3 ± 21.9	31.5 ± 61.2	< 0.01		13.7 ± 11.1	15.1 ± 13.0	35.1 ± 62.6	< 0.01
Whole grains	114.9 ± 96	109.2 ± 70	105.5 ± 78	0.77		107.1 ± 73	114.7 ± 93	107.7 ± 78	0.81		80.3 ± 62.4	113.7 ± 74.3	135.6 ± 97.0	< 0.01		70.1 ± 53.2	100.3 ± 61.1	159.2 ± 98.5	< 0.01
Nutrients																			
Energy (kcal)	2275 ± 754	2547 ± 682	3156 ± 614	< 0.01		2764 ± 785	2471 ± 733	2744 ± 783	0.03		2139 ± 567	2584 ± 683	3254 ± 628	< 0.01		2361 ± 784	2555 ± 724	3061 ± 646	< 0.01
Protein (g/d)	82.0 ± 29.7	95.5 ± 25.4	121.5 ± 26	< 0.01		109.6 ± 32.4	93.0 ± 29.3	96.4 ± 31.1	< 0.01		87.5 ± 28.5	97.1 ± 30.6	114.4 ± 30.1	< 0.01		81.9 ± 27.8	94.2 ± 25.5	122.8 ± 26.9	< 0.01
CHO (g/d)	327.2 ± 121	359.8 ± 121	432.0 ± 98	< 0.01		393.0 ± 129	347.3 ± 114	378.8 ± 119	0.06		295.7 ± 81	355.1 ± 107	468.2 ± 106	< 0.01		331.4 ± 116	360.1 ± 109	427.4 ± 121	< 0.01
Fat (g/d)	77.0 ± 32.1	86.2 ± 27.3	112.7 ± 31.7	< 0.01		92.9 ± 34.3	84.1 ± 29.8	98.9 ± 36.0	0.02		71.6 ± 26.8	92.1 ± 30.5	112.1 ± 31.5	< 0.01		84.4 ± 33.2	87.9 ± 35.6	103.5 ± 29.9	< 0.01
Sodium (g/d)	5.6 ± 3.6	5.7 ± 3.1	7.7 ± 6.0	< 0.01		7.9 ± 6.5	5.6 ± 2.7	5.4 ± 2.8	< 0.01		5.6 ± 4.0	6.0 ± 3.3	7.4 ± 5.8	0.03		8.4 ± 6.5	5.0 ± 1.9	5.6 ± 3.1	< 0.01
Calcium (g/d)	1.0 ± 0.4	1.2 ± 0.5	1.5 ± 0.4	< 0.01		1.5 ± 0.6	1.1 ± 0.3	1.0 ± 0.3	< 0.01		1.1 ± 0.5	1.2 ± 0.4	1.4 ± 0.4	< 0.01		1.2 ± 0.6	1.2 ± 0.4	1.3 ± 0.4	0.05
Fiber (g/d)	24.6 ± 17.0	21.1 ± 10.9	27.0 ± 12.0	0.03		34.0 ± 18.4	20.4 ± 6.7	18.3 ± 6.4	< 0.01		17.9 ± 6.9	21.3 ± 8.8	33.4 ± 17.6	< 0.01		21.2 ± 10.8	21.4 ± 9.6	30.1 ± 17.5	< 0.01

Data are presented as mean ± SD

Abbreviation: CHO: carbohydrate

^*^Obtained from the analysis of variance (ANOVA)

Multivariable-adjusted OR and 95% CI for elevated levels of AGEs and hs-CRP across the tertiles of dietary patterns’ scores are shown in Table 4. A significant positive association was found between adherence to the Western dietary pattern and AGEs (OR: 4.84, 95%CI: 2.29–10.23). This association was significant even after controlling for demographic characteristics, energy intake, DM-related training, physical activity, drug use, and BMI so that individuals in the highest tertile of the Western diet had 4.32 times greater odds for having elevated AGEs compared with those in the lowest tertile (OR: 4.32, 95% CI: 1.86–10.05). No other significant association was seen between dietary patterns and elevated levels of AGEs.

**Table 4:** Multivariable odds ratios and 95% CI for the association between adherence to dietary patterns and laboratory parameters

		“Western” dietary pattern				“Unhealthy” dietary pattern				“Traditional” dietary pattern				“Semi-healthy” dietary pattern		
		T2	T3	P^*^		T2	T3	P^*^		T2	T3	P^*^		T2	T3	P^*^
^a^ High AGEs																
	Crude	2.47 (1.15–5.29)	4.84 (2.29–10.23)	< 0.01		0.92 (0.47–1.81)	1.00 (0.51–1.95)	1.00		1.10 (0.56–2.17)	1.19 (0.60–2.34)	0.60		1.42 (0.71–2.86)	1.92 (0.97–3.82)	0.06
	Model 1	2.51 (1.14–5.53)	4.32 (1.87–9.97)	< 0.01		1.11(0.55–2.23)	0.97 (0.48–1.98)	0.94		0.88 (0.42–1.85)	0.66 (0.29–1.53)	0.34		1.39 (0.68–2.82)	1.47 (0.70–3.11)	0.33
	Model 2	2.56 (1.15–5.67)	4.32 (1.86–10.06)	< 0.01		1.13 (0.55–2.30)	0.96 (0.46-2.00)	0.92		0.93 (0.44–1.97)	0.67 (0.28–1.56)	0.36		1.44 (0.69–3.01)	1.48 (0.67–3.26)	0.36
	Model 3	2.55 (1.15–5.64)	4.32 (1.86–10.05)	< 0.01		1.13 (0.55–2.30)	0.96 (0.46-2.00)	0.92		0.92 (0.43–1.96)	0.67 (0.28–1.57)	0.37		1.44 (0.69-3.00)	1.47 (0.67–3.24)	0.36
hs-CRP > 3 mg/L																
	Crude	2.27 (1.03–4.98)	3.11 (1.43–6.73)	< 0.01		0.71 (0.34–1.44)	0.77 (0.38–1.56)	0.46		1.28 (0.62–2.65)	1.39 (0.68–2.87)	0.36		0.56 (0.28–1.13)	0.36 (0.17–0.76)	< 0.01
	Model 1	2.21 (0.98–4.95)	3.18 (1.34–7.55)	< 0.01		0.75 (0.36–1.56)	0.75 (0.36–1.57)	0.47		1.13 (0.52–2.44)	1.11 (0.47–2.64)	0.83		0.50 (0.25–1.03)	0.24 (0.10–0.55)	< 0.01
	Model 2	2.29 (1.00-5.23)	2.89 (1.20–6.96)	0.01		0.88 (0.41–1.86)	0.93 (0.43–2.01)	0.88		1.16 (0.52–2.55)	1.27 (0.52–3.09)	0.65		0.33 (0.15–0.74)	0.12 (0.04–0.33)	< 0.01
	Model 3	2.39 (1.04–5.49)	2.97 (1.22–7.24)	0.01		0.87 (0.41–1.86)	0.94 (0.43–2.03)	0.89		1.22 (0.54–2.71)	1.25 (0.51–3.06)	0.68		0.33 (0.15–0.74)	0.13 (0.05–0.35)	< 0.01

Data are presented as OR (95% CI)

Abbreviation: AGEs: advanced glycation end products, hs-CRP: High-sensitivity C-reactive protein, DM: diabetes mellitus, BMI: body mass index

^*^P for trend: obtained from binary logistic regression

^a^ The Third tertile of AGEs was considered as high AGEs

The T1 tertile was considered as reference

Model 1: Adjusted for age, gender, and energy intake

Model 2: Additionally adjusted for smoking, duration of diabetes, DM-related training, physical activity, and using DM-related drugs

Model 3: Further adjustment for BMI

Moreover, there was a significant positive association between the Western diet and elevated levels of hs-CRP (> 3 mg/L) (OR: 3.11, 95% CI: 1.43–6.73). After taking potential confounders into account, individuals in the top tertile of the Western diet were 2.97 times more likely to have elevated hs-CRP compared with those in the lowest tertile (OR: 2.97, 95% CI: 1.22–7.24). We also found a significant inverse association between the semi-healthy dietary pattern and elevated hs-CRP levels (OR: 0.36, 95% CI: 0.17–0.76) so that after taking potential confounders into account, participants in the highest tertile of the semi-healthy dietary pattern had 87% lower odds of elevated hs-CRP compared with those in the lowest tertile (OR: 0.13, 95% CI: 0.05–0.35). There was no other significant association between major dietary patterns and hs-CRP levels.

## Discussion

In the current study, we found that adherence to the Western dietary pattern was positively associated with elevated levels of AGEs in type 1 diabetic patients. Moreover, there was a significant positive association between the Western diet and increased levels of hs-CRP, while the semi-healthy dietary pattern was inversely associated with hs-CRP levels. These associations were obtained after taking potential confounders into account. In contrast, we found no significant association between unhealthy/traditional dietary patterns and AGEs/hs-CRP levels among T1DM patients.

Diabetes mellitus is a public health problem around the world [1]. AGEs contribute to the pathophysiology of various diseases such as vascular diseases in diabetes [15, 27]. In addition, AGEs have deleterious effects on gastrointestinal tract through their contribution to markedly altering the gut structure leading to increased intestinal permeability and reduced expression of enteric neurons, as well as to reshaping the microbiota composition [28]. AGEs can be formed in the systemic circulation due to hyperglycemia (endogenous AGEs) [29] and also in the gut (enterically formed FruAGEs) due to unabsorbed fructose which results from intake of unpaired fructose [7]. Therefore, uncontrolled hyperglycemia increases the rate of protein glycation and therefore the production of endogenous AGEs [29]. High-glycemic index (GI) diets, such as the Western diet, increase the severity of hyperglycemia in diabetic patients [30].

In the current study, adherence to a Western dietary pattern containing high amounts of snacks (French fries, chips, and puff), sweets and desserts, pizza, sweetened drinks, potatoes, and eggs was associated with elevated levels of AGEs. Western diet is a fructose- and glucose-rich diet [31]. Fructose stimulates insulin secretion less than glucose and glucose-containing carbohydrates [32]. In addition, the presence of sweetened beverages (a combination of fructose and glucose) in the Western diet can increase the glucose levels in diabetic patients and subsequently enhance the chance of protein glycation [33]. The positive association between the Western diet and hyperglycemia was also confirmed in previous studies [34–36]. However, rapid clearance of plasma fructose after dietary fructose ingestion makes it unlikely that fructose would have time to affect protein glycation significantly [32]. Furthermore, circulating levels of fructose are significantly lower than glucose [37]. Therefore, glucose has a more important role in endogenous protein glycation compared with fructose [32]. In addition, high-fructose diets result in a high amount of fructose in the gut and then increase the production of enterically formed AGEs (FruAGEs) in the presence of free amino acids [7]. This is the “fructositis” hypothesis, which states that intake of foods and beverages with high fructose-to-glucose ratios (> 1:1) promotes the intestinal formation of readily absorbed, pro-inflammatory extracellular, newly identified, fructose-associated AGEs [7]. Based on the DeChristopher et al. study, fruits such as apple, fruit juice, and high-fructose corn syrup (HFCS) have a fructose-to-glucose ratio of > 1:1. In the current study, the Western diet contained a low amount of fruits and fruits juice. In addition, the beverages in the pattern were usually sweetened with sugar or sucrose with a 1:1 ratio of fructose-to-glucose. Therefore, the positive association between the Western pattern and AGEs levels in the current study cannot be mediated by intestinal formation of AGEs. Also, based on the DeChristopher et al. study, fructose may increase the intestinal formation of AGEs when the fructose absorption was not complete. In addition to the role of protein and carbohydrate, the Western diet has a high amount of fat. Li et al. reported that adherence to a high-fat diet was associated with elevated serum and visceral levels of AGEs [38].

Unlike our study, in the Semba et al. study, fast food intake was not associated with elevated serum AGEs [8]. The Semba findings were consistent with the study of Sebeková et al., in which the consumption of high-heat-treated foods (grilled, broiled, and fried meats) was not correlated with AGEs levels in omnivores [9]. Although it is proposed that fast foods such as snacks and pizza contain a high amount of AGEs [8], DeChristopher et al. reported that data on the positive association between fast foods and AGEs levels are limited and inconsistent [7].

It must be kept in mind that sweetened drinks and desserts in Iran are sweetened with sucrose rather than HFCS because Iran does not produce or import HFCS [39]. No studies have assessed the difference between HFCS and sucrose in terms of their effects on circulating AGEs. However, the levels of fructose and glucose are comparable. HFCS contains a higher amount of fructose compared to sucrose [40]. Therefore, sucrose provides a lower amount of unabsorbed fructose in the gut and maybe a lower production of enterically formed AGEs (FruAGEs) compared to HFCS [7]. However, sucrose contains a high amount of glucose and may increase the endogenous production (protein glycation in the blood) of AGEs [40]. Overall, future studies should compare the effects of HFCS and sucrose on AGEs in diabetic patients.

In the current study, we found no significant association between the semi-healthy dietary pattern and serum levels of AGEs in diabetic patients. In the current study, this dietary pattern contains whole grains and white and processed meats. In the Semba et al. study [8], intakes of whole grains and chicken were positively associated with serum levels of AGEs; however, no significant association was reported for intakes of fish and processed meats such as sausage and hamburger. The inconsistency might be explained by the different qualities of whole grain foods across different countries. In Iran, the bran of grains is removed during food processing and therefore limited types of whole grain foods are available to people. In addition, different cooking methods among different nations are another reason for the observed inconsistency.

In this study, adherence to a Western diet was associated with increased levels of hs-CRP in patients with T1DM. The Western diet contained a high amount of snacks (French fries, chips, and puff), sweets and desserts, pizza, sweetened drinks, potatoes, and eggs; all the foods have high inflammatory potential. Consistent with our findings, Lopes et al. reported a positive association between ultra-processed food intake and CRP levels in adult women [41]. Another cross-sectional study also reported such a positive association [42]. It is well-known that fast foods are rich in trans and saturated fatty acids, which increase the concentrations of inflammatory biomarkers [43]. These fatty acids seem to stimulate inflammatory signaling pathways by a process involving toll-like receptor (TLR) 4 and subsequently nuclear factor-kB that increases the expression of several inflammatory genes [43, 44].

We found that adherence to a semi-healthy dietary pattern (high consumption of white meat, whole grains, processed meat, and a low intake of salt) was associated with reduced levels of hs-CRP. In agreement with our results, the Multi-Ethnic Study of Atherosclerosis showed that adherence to a dietary pattern containing a high amount of whole grains and a low amount of sodium was inversely associated with serum concentrations of CRP, IL-6, and homocysteine [45]. In a cross-sectional study, adherence to a prudent pattern characterized by higher intakes of fruit, vegetables, legumes, fish, poultry, and whole grains was associated with reduced levels of CRP [46]. Whole grains contain a high amount of antioxidants, fiber, and magnesium with anti-inflammatory properties [47, 48].

This was the first study that examined the association between major dietary patterns and AGEs/hs-CRP levels among patients with T1DM. However, some limitations should be considered when interpreting our findings. First, our study had a cross-sectional design, which made us unable to draw any causal link between dietary patterns and AGEs/hs-CRP levels in patients with T1DM. Second, we could not perform subgroup analyses based on gender and BMI status due to the low sample size. Third, we controlled for a wide range of potential confounders; however, the potential effects of residual confounders cannot be excluded. Fourth, despite using a validated FFQ for dietary assessment, some degree of measurement errors and misclassification may have occurred, and we cannot entirely exclude this possibility from our study.

## Conclusion

We found that adherence to a Western dietary pattern (high consumption of snacks, potatoes, sweets and desserts, pizza, sweetened drinks, and eggs) was associated with increased levels of AGEs and hs-CRP in patients with T1DM. In addition, adherence to a semi-healthy diet (high consumption of white meat, whole grains, processed meat, and a low salt intake) was associated with reduced levels of hs-CRP in these patients. We found no significant association for other dietary patterns including unhealthy and traditional dietary patterns. Overall, based on our findings, T1DM patients should adhere to a diet containing a high amount of whole grains and a low amount of fast foods, sweets and desserts, sweetened drinks, and salty foods to prevent long-term complications of diabetes. In addition, the intake of white meats, instead of red meats, is recommended. However, these recommendations should be confirmed in well-designed randomized controlled trials.

## Electronic supplementary material

Below is the link to the electronic supplementary material.

Supplementary Material 1: Food grouping used in the dietary patterns

## Data Availability

All the data supporting the findings of this study are available on request from the corresponding author. Due to privacy and ethical restrictions, the data are not publicly available.

## Abbreviations

AGEs: advanced glycation end product
hs-CRP: high-sensitivity C-reactive protein
T1DM: type 1 diabetes mellitus
FFQ: food frequency questionnaire
IPAQ: International Physical Activity Questionnaire
BMI: body mass index
DM: diabetes mellitus
PA: physical activity
HbA1c: hemoglobin A1c
