# Supplementary material for: Association of major dietary patterns with advanced glycation end products and high-sensitivity C-reactive protein in people with type 1 diabetes mellitus
Source: Nutr J. 2023 Jul 26;22:37. doi: 10.1186/s12937-023-00860-x (PMC10369805; doi:10.1186/s12937-023-00860-x)
Supplement: Supplementary file 1 — Supplementary Material 1: Food grouping used in the dietary patterns [file 12937_2023_860_MOESM1_ESM.docx]

**Supplementary Table 1**: Food grouping used in the dietary patterns

| Food group | Food items |
| --- | --- |
| Whole grains | Barley, Sangak, Barbari, Corn and Groats |
| Refined cereals | Lavash, Taftoon, Baguette, Toast, Rice, Spaghetti, Vermicelli, Noodle and Wheat flour |
| Fruits | Cantaloupe, Melon, Canary melon, Watermelon, Pear, Apricot, Cherries, Apple, Peach, Nectarine, Greengage, Fresh fig, Grape, Kiwi, Grapefruit, Orange, Persimmon, Tangerine, Pomegranate, Plum, Strawberry, Banana, Sweat lemon, Lemon, Pineapple and fresh Berries |
| Vegetables | Lettuce, Tomatoe, Cucumber, Sabzi (mixed vegetable), Stewed vegetables, Green sqaush, Pumpkine, Eggplant, Celery, Green pea, Green bean, Carrot, Boiled carrots, Garlic, Onion, fried onions, Cabbage, Sweat pepper, Spinach, Stewed spinach, Turnip and Mushroom |
| Legumes | Lentil, bean, ChickPea, Split pea, Broad bean, Mung bean and Soybean |
| Nuts | Almond, walnut, pistachio, hazelnut, peanut, Sunflower seed and olive |
| Dried fruits | Raisin, Dried berry, Peach leaf, Apricot leaf, Date, Dried fig |
| Low-fat dairy products | Low-fat milk, cheese and low-fat yogurt, Dough(yogurt drink) |
| High-fat dairy products | High fat milk, Chocolate milk, Cream cheese, Strained yogurt, High fat yogurt, Creamy yogurt, Dough(yogurt drink), Curd(kashk), Traditional ice cream, Ice cream, Pizza cheese and Sour cream |
| White meat | Chicken with skin, Chicken without skin, All type of Fish, Tuna |
| Red meats and Organ meats | Beef, Lamb, Ground beaf, Heart, liver, Kidney, tongue, brain, Tripe, Intestine, Viscera and Kale pache |
| Solid oil | Hydrogenated oils, rump, Animal fat, Butter and Margarine |
| Liquid oil | Liquid oil and olive oil |
| Eggs | Eggs |
| Pizza | Pizza |
| Snack | French fries, Chips and Puff |
| Processed meats | All type of Sausages, Kielbasa and Hamburger |
| Sweet drinks | Soft drinks, Apple juice, Orange juice, Cantaloupe juice and Compote |
| Sweets and Desserts | Biscuit, Cracker, Yazdi cake, Cake, Pastry, Cookie, Gaz, Candy, Sugar, Honey, Jam, Sohan, Chocolate, Caramel, Noghl, Rock candy, Halva and Doughnuts |
| Potato | Potato |
| Mayonnaise sauce | Mayonnaise sauce |
| Salt | Salt |
| Tea and coffee | Tea and Coffee |
